# Diphtheria

**Published:** 1860-02

**Authors:** W. Godfrey Dyas

**Affiliations:** Chicago


### ARTICLE 3.

### DIPHTHERIA.

BY W. GODFREY DYAS, M. D., CHICAGO.

[Continued from Page 22.]

In the treatment of all epidemic diseases, the science and practice of therapeutics must be subordinate to the prevailing medical constitution.

The external agents that concur in the formation of the atmosphere, and which are in relation to the animal economy, are collectively termed the *atmospheric constitution.* Now certain modifications are impressed on the organism by these agents either suddenly and violently when they are exaggerated in degree, or more slowly and continuously, though less in degree.

To the former, may be traced the cause of various inflammatory affections of internal organs; in the latter, a less marked, yet more prolonged action, whether accompanied or not by some new element in addition to the usual elements of the atmosphere, may determine successive and special changes in the system. It is to these changes thus effected, that the phrase *medical constitution* is applied. It is the transition state between health and positive disease. A state not clearly expressed, yet notwithstanding seen through the attendant derangement of the digestive organs, oppressed nervous centres and general sense of indisposition that prevail. The whole system is acted on as the result of the slow, gradual and steady operation of a peculiar medical constitution; for not one organ only, but every apparatus of the economy, to a greater or less extent, partakes of an altered state of existence. Thus, what ever the medical constitution may be, we know it by its effects, impressed as they are, more or less, on the greater number of those who come within the sphere of its action. Yet some are much more susceptible of the impression than others. A few peculiarly constituted and some whose organs have been habituated to this abnormal state, may not respond; but they constitute the exception, not the rule. *

Previously to the appearance of cholera in London about 28 years ago, Dr. Prout had found there the oxalic diathesis of unusual occurrence, but when that disease appeared he remarked that the urine of every individual examined, whether in apparent health or otherwise, assumed that peculiar appearance which he had been accustomed to consider as characteristic of the presence of oxalic acid. † Others complained of derangement of the digestive organs, with tendency to diarrhœa; many experienced nothing but *malaise.* These were but several modes of expression, of the prevailing medical constitution, which when fully developed constituted the *epidemic constitution.* Something similar may be seen during an epidemic of scarlatina, when there will be a general predisposition to sore throat, even amongst those who previously had the exanthem.

The modifications of the external world, and their consequent action on the organism are infinite; and if thus infinite, medical constitutions which are but the representatives of this action, will be equally numerous, and thus give rise to as various indications of practice.

Thus the epidemic dysentery in France in 1661, was almost invariably cured by vomits, whereas the same remedy in the dysentery of 1666 was as invariably followed by serious results. *

The typhoid fever epidemic in Ireland between the years 1820—30, admitted of bleeding and depressing doses of antimonials, whereas the same disease when epidemic in that country in 1847, imperatively demanded a treatment the very reverse. ‡

The disease which at present more immediately occupies our attention, is one to which the above prefatory remarks, which reflect the opinions of the distinguished editor of the Gazette Medicale, will especially apply. In some essays and papers on the subject of Diphtheria, we occasionally find wri-

* Gazette Medicale, 1833 † Prout on Stomach and Renal Disease.

‡ Mills on Fever.

ters altogether deny the Diphtheria of Bretonneau's day to be that of a more recent period; evidently not allowing for the distinctive traits of epidemics as they appear at different times and places; and forming a judgment apparently from the result of merely their own individual and therefore limited experience, affect to ridicule the practice of others. The disease is essentially the same as it was then, but accidental circumstances have modified it, and a knowledge of these will be the key to its successful treatment.

Diphtheria has been seen under a croupal form; has been also seen in its most malignant type without croup; and again where little more than *malaise* and slight soreness of throat, with indistinct manifestation of false membrane were the symptoms. Now it will be invariably found that the more croupal the symptoms, the less asthenic will be the disease; whereas in the malignant form uncomplicated with extension to the larynx, the contrary obtains. This will, to some extent explain Bretonneau's advocacy of calomel in Diphtheria. Dr. Meigs approved of it. In addition, he recommended general bleeding, and yet he had considerable success. Others have also used calomel and recorded their approval of it. We must suppose that in these several instances, each particular medical constitution of the day carried with it peculiar therapeutic indications which experience alone taught physicians to fulfill.

As Diphtheria has appeared of late, and to such we mean to confine our remarks, we cannot think favorably of the administration of mercury. Even before the disease explodes, it will have been preceded by a depressed state of the system, an altered condition of the blood and a predisposition in the organs to receive a still further morbid impression; for it must be remembered, that a previous medical constitution had been in operation, diminishing the capability of resistance on the part of the system, and thus preparing it to admit of disease. In such a state of things, calomel unless it positively neutralizes some morbid material, or counteracts some morbid action in the blood, is incapable of effecting anything but mischief. We are not aware of any writer recommending its administration in Diphtheria with either view. Nor does the record of cases detailed of late lead us to infer that such effects followed its exhibition. The most that has been proposed, is to change the action of the capillary system and thus to control the formation of the false membrane, or to lessen plasticity of the blood, and in this way to rob it of the material exuded on the surface of the mucous membranes.

But a *direct* action effected by mercury on the general capillary and absorbent systems, is though a long cherished yet an erroneous opinion. Mercury *indirectly* promotes absorption and counteracts effusion by impoverishing the blood and depriving it of its fibrin, albumen and corpuscles. Thus, the material of which the effusion consists is diminished, and if the latter had been already poured out, the absorbents, in order to restore to the blood an ingredient, of which the system feels the want, will, in obedience to some law of adaptation, take up nutritive matter wherever they can find it.

The exhibition of mercury in Diphtheria with this view, is based on a supposed analogy to croup. The two diseases, though having some features in common, are however essentially different, and besides, the points of difference are so striking and involve such important considerations, as altogether to forbid similar treatment. In croup, there is increased fibrin in the blood, not a putrid crasis nor a tendency to it, as in Diphtheria; in the one, there is the sthenic type of fever, in the other, a typhoid, or even no fever whatever; the former is not preceded by a depressed, nor followed by a cachectic state; these conditions more or less usher in and supervene on the symptoms of the latter. Alkalies, salines, quinine and iron, have each their advocates in Diphtheria. Alkalies are recommended with the view of diminishing the fibrin of the blood; but it has been already shown that the disease may be complicated with scurvy, an affection in which there is a surplus of alkalies, with a deficiency of fibrin in the circulation; and nevertheless Diphtheria has declared itself, thus falsifying the theory on which the recommendation is based.

Chlorate of Potash is a favorite remedy with many. Generally employed empirically or on the supposition that there is here a deficiency of the oxygen required for the maintenance of the vital force, and that a necessity exists for raising the amount to the normal standard. A few imagine that the potash when set free from its state of combination, dissolves the fibrin, from which they apprehend all the danger; but, from what has been already stated under the head of alkalies, it is evident this opinion cannot be maintained. The former view is advocated by some who have, as they conceive, derived benefit from its exhibition in affections allied to this by their more prominent general symptoms, and in connection with which, typhus fever and consecutive anæmia indicated some loose analogy. In order to explain its *modus operandi*, they suppose the Chlorate of Potash having been absorbed and decomposed in the blood, yields out of 245 grains, about 149 grains of the Chloride of Potassium, one of the constituents of blood; and 96 grains or 281 cubic inches of oxygen. This oxygen in its nascent state, in the act of separation, is in the most favorable condition to combine with matter within the sphere of its action. As it is assumed therefore, that there is in Diphtheria a deficiency of oxygen in the blood, it is conjectured that this deficiency is thus supplied. Perhaps after all, it is very much a matter of conjecture how Chlorate of Potash acts on the human organism, and on the whole it is doubtful if it possesses a great deal of therapeutic value in Diphtheria. It may be prescribed in combination with some bitter infusion, whether cascarilla, gentian or bark, in doses of from 10 to 30 grains, according to the age of the patient.—Mr. Lambden (*Lancet, Nov.* 1858) prescribes Chlorate of Potash in combination with hydrochloric acid, so as to have free chlorine in solution. He directs ʒ ii of Chlorate of Potash to be finely powdered, on which ʒ i of diluted hydrochloric acid is to be poured. As soon as the whole powder assumes a yellow color and the fumes of chlorine begin to arise, ℥ viii of water should be instantly added, by which the escape of chlorine is prevented. This may be given in the dose of a tablespoonful every one, two or three hours, according to the urgency of the symptoms.

Emetics are occasionly serviceable, especially in young children, at the period of invasion of the disease and when the false membrane extends to the trachea. They should never be omitted when together with fever, there are foul tongue, want of appetite, and nausea early in the complaint. For very young children, perhaps some preparation of squill will be the best emetic in Diphtheria. Owing to their depressing effects, neither antimony nor ipecacuanha should be used. Alum as employed by Dr. Meigs, in doses of a teaspoonful every ten, fifteen or twenty minutes, is a certain and speedy emetic.—The Subsulphate of Mercury (*Turbith Mineral.*) recommended by Dr. Hubbard of Hallowell, Maine, is a prompt and certain means of producing emesis. It never induces catharsis, and is not followed by prostration. It may be given in doses of from two to three grains to a child of two years, and repeated every ten or fifteen minutes until vomiting takes place.—Both alum and turbith mineral are well suited to children above one year old. They are simply irritant, and like others of the same order of emetics, cause vomiting without depressing the nervous system, as antimony and ipecacuanha invariably do, from the sedative effect they exert on the vagus nerve.

Independently of their removing morbid secretions, emetics seem to effect some change in the nervous system that controls and directs the physiological transformations of the blood, and when these transformations are abnormal, the deviation from the healthy state may be corrected or modified by some impression resulting trom such change. But it is on stimulants and tonics that the chief reliance, at least in our present state of knowledge, must be placed in the treatment of Diphtheria; topical applications to the fauces being at the same time em ployed as auxiliary to the general treatment. From the commencement of the attack, wine and nourishment should be liberally supplied. This cannot be always easily effected, as the child dreads the pain caused by deglutition. Then recourse must be had to enemata of broth conjoined with quinine and brandy. An enema of two ounces of strong beef tea with a tablespoonful of port wine may be given every second hour, and in the same way from three to five grains of Sulphate of Quinine every fourth hour. These quantities are adapted to children of from two to four years of age. Some practitioners depend more on quinine than on iron. Unfortunately in a great majority of severe cases scarcely an opportunity is afforded to develop the good effects of either, as under every circumstance they are both slow in manifesting their action.—Some cases of Diphtheria appear rather injured than benefited by quinine. When the tongue is foul and the stomach irritable, it is better to withhold it, or should it have been begun with, to suspend it, than persist in its administration. Loss of appetite, soft compressible pulse, tremulous tongue, languor and subdued expression, are indications for its exhibition. When a foul tongue and nausea forbid its immediate use, an emetic should be at once given, which will be likely to prepare the system for its reception. The best tonic perhaps that can be used in this complaint, is the bitter wine of iron as prepared by Mr. Thompson. It contains one grain of Wetherell's precipitated extract of bark and two grains of citrate of iron in a teaspoonful of sherry wine. Some prefer the tincture of the sesquichloride of iron in doses of ten to fifteen drops every third or fourth hour.

Whilst treating a case of Diphtheria, it must not be forgotten how frequent a sequela of this disease anæmia proves. By bearing this in mind we are possessed of the key to the management of those several consecutive disorders that betray derangement of the circulation and nervous system. Purpura, dropsy and paralysis may still have to be encountered, and by no means can they, so efficiently, as by preparations of iron. In whatever the *materies morbi* of the disease may consist, it has at least one action easily appreciated by its results, it invariably diminishes the hæmatosin of the blood below its normal standard, and lessens the amount of the blood-corpuscles. Now iron is essential to the chemical constitution of hæmatosin. These considerations lead to the conclusion that as a part of the morbid process consists in reducing the amount of a necessary constituent of the blood, the supplying this constituent may, if it cannot altogether prevent evil, at least prevent its being fully developed or favorably modify it in its development. It may thus be said that in Diphtheria iron produces an action contrary to a diseased one, yet there is no reason to suppose that it directly neutralizes the morbid action.

In treating a case of Diphtheria, local applications should by no means be omitted. Nevertheless, it ought not to be forgotten, that the disease is constitutional, and that the exudation on the fauces is merely a local manifestation. Still local applications will retard and even limit the extension of the false membrane, which by plugging up the respiratory passages may cause death. Thus they are of use, but otherwise, they are unavailing in influencing the progress of the general disease. An unmerited importance has been given them by writers, who frequently assign them in this disease a higher place than they allow to the general treatment. The principal topical remedies are the nitrate of silver, hydrochloric acid, tincture of sesqui-chloride of iron, and the chlorides of lime and soda. Nitrate of silver ought never to be applied except in solution; its application in substance has occasionally had most serious consequences; perhaps a solution of 30 grains to the ounce will give whatever benefit is to be derived from it. Hydrochloric acid may be used undiluted, or where young children are the subjects of treatment, the acid may be mixed with an equal quantity of honey. Monsell's salt of iron is a safe and manageable topical application and has been found most useful.

The paralysis so frequently consequent on Diphtheria is dependent perhaps as much on the absence of what is necessary to the due excitation of the nervous centres, as on the superadded influence of some positive materies morbi. It bears a striking resemblance to what is termed chlorotic paralysis so well described by Sandras, (*Gazette des Hopitaux, Janvier*, 1855,) and like it does not arise from a lesion of the nervous tissue. A knowledge of this fact guides us to correct therapeutic indications. The blood must be supplied with its normal proportion of hæmatosin, and this is to be effected through the agency of preparations of iron. Generous and supporting diet is not to be omitted. Sulphur baths, electricity, preparations of zinc, and valerian, are occasionally valuable auxiliaries.

In conclusion, it is to be observed that there is scarcely any case of this, or indeed of any other disease, which does not from its peculiar features require modifications of treatment that can only be determined by the judgemnt of the person in attendance, but we lay down the following summary of treatment as that which will be found most generally applicable in a large majority of cases. Early in the attack the administration of an emetic—then a gentle purgative of some mild preparation of mercury and rhubarb, and the application of a 30 grain solution of nitrate of silver to the fauces. Immediately after the bowels shall have been moved, tincture of sesqui-chloride of iron should be commenced with, alternating the doses with port wine and beef tea, and at the same time the throat may be enveloped in flannel steeped in some stimulating embrocation. Some prefer a large poultice of chamomile flowers, whilst others object to all moist applications of this kind. Under no circumstances whatever should leeches or blisters be applied, as they not only tend to still further depress the system, but are apt to be followed by cutaneous Diphtheria.

